# A Review of Curcumin in Chronic Disease Management: Anti‐Inflammatory Pathways, Antioxidant Activity, and Therapeutic Advances

**DOI:** 10.1155/jnme/9985642

**Published:** 2026-05-18

**Authors:** Sammra Maqsood, Farhang Hameed Awlqadr, Iffat Ullah, Muhammad Tayyab Arshad, Humaira Parveen, Sayeed Mukhtar, Emmanuel Laryea

**Affiliations:** ^1^ National Institute of Food Science and Technology, University of Agriculture Faisalabad, Faisalabad, Pakistan, uaf.edu.pk; ^2^ Food Science and Quality Control, Halabja Technical College, Sulaimani Polytechnic University, Sulaymaniyah, Iraq, spu.edu.iq; ^3^ Department of Pharmaceutical Chemistry, Faculty of Pharmaceutical Sciences, Prince of Songkla University, Hat Yai, 90110, Songkhla, Thailand, psu.ac.th; ^4^ Drug Delivery System Excellence Center, Faculty of Pharmaceutical Sciences, Prince of Songkla University, Hat Yai, 90110, Songkhla, Thailand, psu.ac.th; ^5^ Functional Food and Nutrition Program, Center of Excellence in Functional Foods and Gastronomy, Faculty of Agro-Industry, Prince of Songkla University, Hat Yai, 90110, Songkhla, Thailand, psu.ac.th; ^6^ Organic and Medicinal Chemistry Research Lab, Department of Chemistry, Faculty of Science, University of Tabuk, Tabuk, 71491, Saudi Arabia, ut.edu.sa; ^7^ Department of Food Science and Technology, Kwame Nkrumah University of Science and Technology, Kumasi, Ghana, knust.edu.gh

**Keywords:** cardiovascular disorders, curcumin, gut microbiota, inflammation, NF-κB pathway

## Abstract

The medicinal properties of *Curcuma longa* L. (turmeric) have long been recognized, especially in traditional medicine, where it has been used to treat diverse ailments for thousands of years. The bioactive polyphenol responsible for the health benefits associated with *C. longa* is curcumin, which offers a variety of health advantages, particularly, anti‐inflammatory properties. This review discusses the molecular mechanisms underlying the anti‐inflammatory properties of curcumin, focusing on its interaction with significant inflammatory signaling pathways, modulation of the gut microbiota, and antioxidant activity. The therapeutic potential of curcumin is rooted in its ability to reduce enzymes such as COX‐2 and LOX, downregulate proinflammatory cytokines, and block the NF‐κB pathway. In addition to its ability to decrease oxidative stress, a primary mediator of inflammation, curcumin also functions in scavenging reactive oxygen species (ROS) and activating antioxidant defense systems. This review further highlights the clinical relevance of curcumin in chronic inflammatory conditions including rheumatoid arthritis, inflammatory bowel disease, and cardiovascular disorders. Despite its encouraging therapeutic potential, curcumin bioavailability remains a major problem, prompting advancements in drug administration methods. Further research is needed to improve its formulations, study how curcumin synergizes with other treatments, and conduct clinical studies.

## 1. Introduction

The golden yellow rhizome of *C*. *longa* from Zingiberaceae, cultivated for both culinary and medicinal purposes has been of interest since ancient times. Because of its bright color and savory flavor, *C. longa*, being used in food, is a functional food based on the criteria of utilization in areas of health and well‐being, apart from the fields of gastronomy [1, 2]. *C. longa* has been used for centuries; it is native to the Indian subcontinent, where it forms an integral part of Siddha and Ayurvedic medicines. Some of the various applications of *C. longa* include the treatment of respiratory disorders, skin disorders, gastrointestinal problems, and inflammation [3, 4].

The cultural relevance of *C. longa* is further emphasized by its utilization in rituals and spiritual activities, where its curative properties are tied to holistic health techniques [5]. It has been used in ancient Middle Eastern and African systems for treating gastrointestinal and hepatic conditions and in traditional Chinese medicine for pain and circulation‐related disorders [6].

The cross‐cultural use of *C. longa* further underlines its versatility and century‐long history in traditional medicine. *C. longa* has only recently been designated as a functional food based on scientific findings. In addition to fulfilling nutritional requirements, functional foods enhance well‐being in other ways, typically by adding bioactive compounds [7]. *C. longa* contains the polyphenolic compound curcumin, which is highly effective. Evidence has demonstrated that it exerts antioxidant, anti‐inflammatory, and antibacterial effects [8]. Therefore, it acts as a link between the traditional and conventional cooking methods. This is attributed to the increasing awareness of the use of natural remedies in Western nations, where a larger emphasis has been placed on the use of *C. longa* in teas, nutraceuticals, and health supplements over the past decades [9, 10]. Its strength in functional food production is significant because of its ability to treat chronic lifestyle diseases, including diabetes, cardiovascular issues, and neurodegenerative disorders, compatible with the current diet trend [11]. While the traditional use of *C. longa* has been largely empirical, modern scientific research has begun to systematically validate these age‐old claims by identifying curcumin as the principal bioactive compound responsible for its therapeutic effects [12, 13].

Curcumin modifies important molecular pathways that are crucial regulators of inflammation through its biochemical versatility to exhibit an impressive array, including cyclooxygenase‐2 (COX‐2), inducible nitric oxide synthase (iNOS), and nuclear factor‐kappa B (NF‐κB) [1, 14]. Moreover, because curcumin can scavenge free radicals and boost endogenous antioxidant systems, it effectively reduces oxidative stress [15, 16]. Although curcumin has poor solubility and rapid metabolism, its bioavailability remains a major problem [17].

New delivery vehicles, such as phospholipid complexes, liposomes, and nanoparticles, are currently being researched to improve their absorption and therapeutic potential [8, 18]. For instance, the incorporation *C. longa* into the regular diet of South Asia has been another dual use of food and medicine. The health benefits of recipes such as curries, herbal teas, and drinks such as *C. longa* milk have long been recognized [5]. The versatility and durability of spices are manifested in their application to modern diets and wellness trends [19]. Moreover, scientific research on the culinary application of *C. longa* has promoted its use as a functional food because it explains how the compound prevents damage to the intestinal mucosa and inflammation [20]. Therefore, the interaction between innovation and tradition maintains *C. longa* for the treatment of modern medical conditions. Curcumin is responsible for the bright yellow color of the spice and is involved in most of its pharmacological properties, including anti‐inflammatory, antioxidant, antibacterial, and anticancer effects [1, 4, 21].

Curcumin can interact with a broad range of molecular targets within the body owing to its polyphenolic nature. It can penetrate cell membranes and interfere with cellular functions because of its hydrophobicity [15]. Its rapid metabolism and low solubility in water limit its bioavailability even though it is potent. Nanoparticles and liposomes, two examples of improved delivery technologies, have been designed to enhance the therapeutic index of drugs by addressing this problem [8, 22]. Curcumin acts as an anti‐inflammatory agent by inhibiting the NF‐κB complex, which is responsible for the production of inflammatory cytokines. Prasad and Aggarwal [1] and Joe, Vijaykumar, and Lokesh [23] reported that curcumin inhibits NF‐κB, which in turn reduces the production of proinflammatory mediators such as interleukins (IL‐6, IL‐1β, and tumor necrosis factor‐alpha [TNF‐α]).

Sharifi‐Rad et al. [6] reported that inflammation‐related disorders such as arthritis, IBD, and heart diseases can be treated with curcumin. Natural antioxidant enzymes catalase and superoxide dismutase (SOD) are increased in activity by curcumin, thus acting as efficient free radical scavengers. It is also known to prevent lipid peroxidation, as reported by Akter et al. [15] and Alrawaiq & Abdullah [16], preventing oxidative damage by shielding cellular structures against the aforementioned neurological illnesses and protecting against diseases such as Parkinson’s disease (PD) and Alzheimer’s disease (AD) [2].

Curcumin also exhibits antibacterial properties against several viruses, fungi, and bacteria. It acts efficiently against antibiotic‐resistant bacterial strains because it disrupts microbial cell membranes and inhibits biofilm production [19]. According to Zeeshan et al. [3], curcumin is a potential treatment for infectious diseases. Curcumin has been reported to have anticancer effects owing to its ability to intervene in several signaling pathways, including p53 and PI3K/AKT. It has been shown to be effective against breast, colon, and prostate malignancies by inducing apoptosis in cancer cells, suppressing metastasis, and inhibiting angiogenesis [14, 24].

Owing to its safety profile, it can be used as an adjuvant in cancer therapy [11]. Its anti‐inflammatory and antioxidant properties have been linked to its neuroprotective effects. Curcumin has been shown to cross the blood–brain barrier and modulate processes that cause amyloid plaque formation, which is a characteristic of AD. Curcumin also supports cognitive health through synaptic plasticity [22, 25]. Although the biological relevance of curcumin is well established, its poor bioavailability hinders its practical applications. Research has focused on enhancing its pharmacokinetics through innovative formulations, such as metal complexes and curcumin conjugates, to improve its stability and absorption [26, 27].

This review aims to elucidate the therapeutic potential of curcumin in reducing chronic inflammation associated with many diseases, such as metabolic syndromes, cardiovascular disorders, neurodegenerative diseases, and arthritis, by discussing its interaction with transcription factors, enzymes, and inflammatory mediators. This review also discusses updates in curcumin delivery systems intended to overcome bioavailability problems, allowing maximum utilization in functional foods, nutraceuticals, and clinical settings.

## 2. Biochemical Properties of Curcumin

**FIGURE 1:**
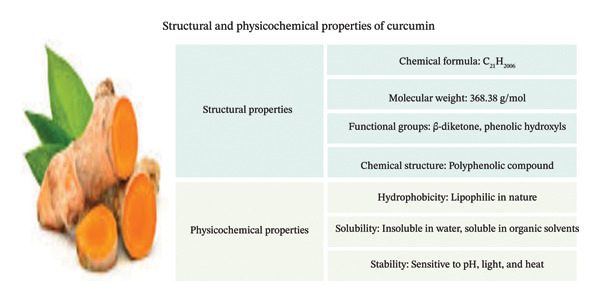
The molecular structure and major physicochemical properties of curcumin that govern its stability, solubility, and bioavailability.

*C*. *longa* consists of rhizomes, and curcumin is one of the principal polyphenols. This type of substance has been extensively studied, because its chemical structure and functional groups support a wide spectrum of biological functions. This makes it valuable as part of traditional and modern medication because of its chemical makeup, which is crucial for its anti‐inflammatory, antioxidant, and therapeutic properties. Based on its chemical structure, curcumin is a diferuloylmethane or symmetrical bis‐α, β‐unsaturated β‐diketone. The methoxy (–OCH_3_) and hydroxyl (–OH) groups were positioned at designated locations on each of the two aromatic (phenolic) rings. These binary rings were linked by a seven‐carbon conjugated chain with a diketone group [26]. The biological activity of curcumin and its distinguishing bright yellow color instigate this conjugated structure. The improved reactivity of this linker with free radicals and macromolecules found in the intracellular environment stems from its double bonds in the middle, arranged in the trans form, to alleviate other molecules (Figure 1) [23]. Figure 1 illustrates the molecular structure and major physicochemical properties of curcumin determine its stability, solubility, and bioavailability.

The diketone group is chiefly significant because it occurs in a form of symmetry between the keto and enol tautomers, as the enol form is more constant in solution. This structural feature improves the capacity of curcumin to interact with nucleophilic sites in proteins and enzymes and chelate metal ions (such as Fe2+ and Cu2+) and form robust hydrogen bonds [22]. These connections form the basis of the antioxidant and anti‐inflammatory actions of curcumin. The key nutritional components of curcumin and their biological significance in health and disease prevention are presented in Table 1.

**TABLE 1:** Key nutritional components of curcumin and their biological significance, highlighting major nutrients and associated biological activities that contribute to its health‐promoting effects.

Component	Composition	Effect	Reference
Carbohydrates	∼5–10% of turmeric rhizome by weight	Curcumin is not a carbohydrate but part of turmeric’s starch component.	[28]
Proteins	∼6–7% of turmeric rhizome by weight	Proteins contribute to the overall nutritional profile of turmeric, but curcumin is not a significant source of protein.	[22]
Fats	∼5% of turmeric rhizome by weight	Turmeric contains essential fatty acids, but curcumin’s fat content is negligible.	[29]
Curcumin (bioactive polyphenol)	∼3–5% of turmeric rhizome weight	Curcumin is the primary active ingredient responsible for turmeric’s medicinal effects.	[29, 30]
Polyphenols	∼1–2% of turmeric rhizome	Curcumin is the main polyphenol in turmeric, exhibiting antioxidant and anti‐inflammatory properties.	[22]
Essential oils	∼3–6% of turmeric rhizome	It contains compounds like turmerone, which contribute to anti‐inflammatory and antimicrobial effects.	[31]
Fibers	∼20% of turmeric rhizome	Dietary fiber in turmeric aids digestion, although curcumin does not significantly contribute to this.	[22]
Minerals	Potassium, magnesium, calcium, iron (trace amounts)	These minerals support general health and complement curcumin’s therapeutic actions.	[29]
Vitamins	Vitamin C, vitamin E (trace amounts)	Minor contribution to antioxidant defense, enhancing curcumin’s effects.	[22]
Turmerones (α, β)	∼0.5–1% of turmeric rhizome	Essential oils that exhibit neuroprotective and anti‐inflammatory properties.	[29]
Demethoxy curcumin	10%–20% of curcumin content	A curcumin analog with similar but more potent anti‐inflammatory effects.	[31]
Bisdemethoxy curcumin	∼10–20% of curcumin content	A derivative of curcumin that enhances antioxidant and anticancer activity.	[22]
Phytosterols	β‐Sitosterol, stigmasterol (trace amounts)	Phytosterols contribute to anti‐inflammatory and cholesterol‐lowering effects.	[28]
Saponins	∼1–2% of turmeric rhizome	Saponins support immune function and may have anticancer properties.	[32]
Flavonoids	∼0.5% of turmeric rhizome	Antioxidant and anti‐inflammatory effects, enhancing curcumin’s bioavailability.	[31]
Resins	∼10–15% of turmeric rhizome	Contribute to turmeric’s anti‐inflammatory, antimicrobial, and antioxidant effects.	[29]
Bitter compounds	*Curcuma amarus*, curcuma resin	Enhance digestive health by stimulating bile production and improving digestion.	[30]

### 2.1. Functional Groups and Their Biological Significance

#### 2.1.1. Hydroxyl (–OH) Groups

The antioxidant properties of curcumin are mainly attributed to its phenolic hydroxyl group. Rendering Roman et al. [27]; curcumin inhibits free radical chain procedures by transferring hydrogen atoms to neutralize reactive oxygen species (ROS) and reactive nitrogen species (RNS). This radical scavenging capability protects proteins, lipids, and DNA from oxidative impairment, in contrast to chronic illnesses, such as cancer and neurological illnesses.

#### 2.1.2. Methoxy (–OCH_3_) Groups

Methoxy substituents on the aromatic rings increased the electron‐donating capacity of curcumin, thereby enhancing its reactivity toward free radicals and improving its free radical scavenging efficiency. These methoxy groups enhance the lipophilicity of curcumin, which is critical for its bioactivity and cellular accumulation and facilitates its incorporation into the lipid bilayers of cell membranes [33].

#### 2.1.3. Diketone (α, β‐Unsaturated β‐Diketone) Group

One of the critical structural components permitting curcumin to interact with its frequent cellular targets is its diketone moiety. This permits curcumin to covalently bind to cysteine residues of enzymes and signaling proteins, thus obstructing its action. These interfaces are vital for understanding how curcumin suppresses proinflammatory mediators, such as NF‐κB and COX‐2, and how it subsidizes the apoptotic pathway in cancer cells [34].

#### 2.1.4. Conjugated Double Bonds

Curcumin is resilient to chemical responses and absorbs UV–visible light because of its conjugated nature, which comprises the change in double bonds in the linking area. Curcumin is employed as a photosensitizer in photodynamic therapy to generate singlet oxygen, a reactive form that can target cancer cells [22]. Curcumin employs conjugated bonds to regulate lipid peroxidation and maintain the cellular redox balance in equilibrium.

## 3. Biological Implications of Curcumin’s Structure

Several organic possessions are accredited to curcumin’s exclusive arrangement of functional clusters.

### 3.1. Antioxidant Activity

The conjugated double bonds and phenolic hydroxyl groups present in curcumin render it a potent free radical scavenger and oxidative stress inhibitor in cells. This property is particularly useful for inhibiting oxidative damage, which is a significant constituent of most diseases and disorders [33].

### 3.2. Anti‐Inflammatory Action

It interacts with transcription factors AP‐1 and NF‐κB to alter the expression of inflammation‐related genes at the transcriptional level from inflammation‐related genes [35]. The alpha‐, beta‐unsaturated ketone group from the structure of curcumin, which can undergo Michael‐type addition to sulfhydryl groups to produce covalent protein interactions [26], causes marked inhibition of enzymes, including COX and LOX.

### 3.3. Metal Chelation

Through the diketone group, curcumin chelates metal ions that decrease the availability of catalytic transition metals, such as copper and iron, thus suppressing Fenton reactions, which produce toxic hydroxyl radicals. This metal‐chelating ability applies to diseases, such as AD, in which the development of amyloid plaques is supported by metal dyshomeostasis [27].

### 3.4. Modulation of Signaling Pathways

Curcumin can modulate several cellular signaling pathways because of its structural flexibility. These pathways include PI3K/Akt, JAK/STAT, and MAPK pathways. As curcumin regulates cell proliferation, survival, and apoptosis, these interactions have demonstrated the potential of curcumin as an anticancer agent [22].

### 3.5. Bioavailability and Metabolism of Curcumin

Although curcumin possesses strong biological activities, it is not extensively used in medicine because of its low bioavailability. The reasons for this limitation include rapid metabolism, poor absorption, and rapid systemic clearance following ingestion [26]. To achieve the full therapeutic potential of curcumin, it is important to determine its metabolic pathways and bioavailability. Owing to its hydrophobicity, curcumin is insoluble in stomach acid. It is highly insoluble in water, which significantly hinders its entry into the bloodstream [33].

Curcumin undergoes extensive metabolism in the intestinal mucosa and the liver after absorption. The circulating amount of the active form is reduced because it is primarily metabolized into water‐soluble glucuronides and sulfates [23]. Curcumin loses even more of its therapeutic potential because its metabolic by‐products are eliminated from the body at a very fast rate. The efficacy of curcumin is limited by its short half‐life in the plasma [34]. Liver enzymes reduce curcumin to dihydrocurcumin and tetrahydrocurcumin (THC). Although not as much as that of the parent substance, these metabolites have some bioactivity and aid in the pharmacological effects of curcumin [27]. Conjugation reactions are the primary metabolic transitions that result in the formation of curcumin glucuronides and sulfates. Bile and urine are the means of excretion of water‐soluble conjugates are bile and urine [22]. Piperine found in black pepper inhibits glucuronidation, elevating the plasma concentrations and bioavailability of curcumin [33].

The solubility and stability of curcumin are enhanced by nanoparticles, liposomes, and micelles, which further increase its absorption and systemic distribution [36]. Chemical analogs of curcumin with modified functional groups have been synthesized to enhance metabolic stability and biological efficacy [37]. Curcumin increases the intestinal absorption and lipophilicity of foodstuffs when amalgamated into emulsions or merged with lipids [38]. Curcumin has great medical prospects, but its main challenge is bioavailability. To overcome these limitations and augment the health benefits of curcumin, new delivery systems, formulation methods, and coadministration with bioenhancers such as piperine are being examined [22, 26].

Curcumin is extremely biologically active owing to its chemical arrangement, but its bioavailability is low owing to its hydrophobic nature and low water solubility [39]. Its rapid metabolism in the intestine and liver also decreases its systemic accessibility and therapeutic effectiveness [34]. Coadministration of curcumin with piperine, a bioenhancer derived from black pepper, increases the availability of curcumin by inhibiting glucuronidation and enhancing intestinal permeability, resulting in improvements in bioavailability in humans and animal models by 2000% and 154%, respectively [40]. However, formulation‐based strategies appear more effective, as biodegradable nanoparticles loaded with curcumin have shown at least a ninefold increase in oral bioavailability compared to piperine–curcumin combinations in animal studies, emphasizing the enhanced capacity of nanoscale carriers to shield curcumin from degradation and improve the kinetics of absorption [41].

Structural analogs, nanoparticles, and liposomes are ongoing developments in formulation approaches that aim to overcome these limitations and improve the stability and bioactivity of curcumin [33]. Direct reasons for the disparate biological properties of curcumin can be elucidated by its molecular structure and functional groups. The medicinal, antioxidant, and anti‐inflammatory activities are reinforced by the existence of conjugated double bonds, α, β‐unsaturated diketone moiety, phenolic hydroxyl, and methoxy groups. Despite the long‐standing bioavailability problems, further research into new formulations and structural modifications could extend the therapeutic applications of curcumin. Curcumin is a valuable therapeutic agent for a variety of disorders because it is metabolically flexible and is involved in cancer and inflammatory disorders. Table 2 outlines the chemical structure and functional groups of curcumin, its bioavailability characteristics, its metabolic fate in the human body, and the principal challenges affecting its clinical translation.

**TABLE 2:** Key structural features and functional groups of curcumin, together with its oral bioavailability profile, metabolic pathways in the human body, and the major physicochemical and biological barriers limiting its systemic availability.

Topic	Detail	Reference
Chemical structure and functional groups of curcumin	Curcumin has a biphenyl structure with two methoxy groups (OCH_3_) attached to the aromatic rings. It also has a β‐diketone functional group. These functional groups contribute to its antioxidant, anti‐inflammatory, and anticancer properties.	[25]
	The curcumin molecule’s functional groups (hydroxyl, methoxy, and carbonyl) enable it to interact with various molecular targets, modulating multiple signaling pathways.	[27]

Bioavailability of curcumin	Curcumin has low bioavailability due to poor absorption, rapid metabolism, and rapid elimination. Various methods, such as coadministration with piperine, have been explored to enhance its bioavailability.	[15]
	Bioavailability is further improved by nanotechnology and encapsulation techniques such as liposomal curcumin or curcumin‐loaded nanoparticles, which protect the compound from degradation in the body and allow targeted delivery.	[22]

Metabolism of curcumin in the human body	Curcumin undergoes rapid Phase I and Phase II metabolism in the liver, including reduction, conjugation with glucuronic acid, and sulfation. These metabolites are then excreted via urine and bile.	[23]
	Curcumin metabolites, such as tetrahydrocurcumin and curcumin glucuronide, have been shown to have bioactive properties that contribute to curcumin’s overall therapeutic effects.	[33]
	The liver, intestine, and gut microbiota play crucial roles in curcumin metabolism, influencing its systemic distribution and bioactivity.	[37]

Challenges in curcumin’s bioavailability	Curcumin’s poor solubility and high metabolism contribute to its low bioavailability. Strategies like curcumin with fats or surfactants have improved its absorption and therapeutic effectiveness.	[26]
	Some formulations aim to prevent first‐pass metabolism using nanoparticles or liposomal formulations, enhancing curcumin’s tissue penetration.	[22]

Impact of curcumin’s chemical structure on biological activity	The presence of phenolic and methoxy groups is essential for curcumin’s biological activity, as these groups allow it to scavenge free radicals, suppress inflammation, and modulate various enzymes involved in cancer cell proliferation.	[25]
	Modifications to the structure of curcumin (e.g., the addition of metal complexes or functionalization) can improve its bioactivity and targeting capabilities for specific therapeutic applications such as cancer therapy.	[26]

## 4. Mechanisms of Inflammatory Modulation

*C. longa*, which contains the active constituent curcumin, has gained considerable attention owing to its ability to modulate inflammation. Curcumin downregulates proinflammatory mediators by inhibiting multiple inflammatory signaling pathways, leading to the reduced production of key inflammatory cytokines and enzymes. This discussion is detailed for curcumin owing to its anti‐inflammatory activity.

### 4.1. Interaction of Curcumin With Inflammatory Pathways

#### 4.1.1. NF‐κB Signaling Inhibition

The signaling cascade is referred to as the NF kappa‐light‐chain‐enhancer of activated B‐cell inflammatory reactions. NF‐κB activation triggers its entry into the nucleus and promotes the transcription of adhesion molecules, enzymes, and proinflammatory cytokine‐encoding genes. Curcumin constrains upstream kinases that phosphorylate IκB proteins and consequently overwhelms NF‐κB activity. According to Edwards et al. [42] and Memarzia et al. [43], this inhibition hinders inflammation by preventing the interruption of IκB by NF‐κB and its subsequent movement to the nucleus. Studies have revealed that curcumin decreases NF‐κB activity in cell culture prototypes, which abridges the expression of other inflammatory cytokines, such as IL‐6 and TNF‐α.

#### 4.1.2. Downregulation of Proinflammatory Cytokines

The proinflammatory cytokines required to tolerate inflammation in both acute and chronic situations are TNF‐α, IL‐6, and interleukin‐1 beta (IL‐1β). To constrain the production of these cytokines, curcumin is restricted to signaling pathways, such as NF‐κB and STAT3. Curcumin has also established potential in the management of inflammatory diseases such as inflammatory bowel disease and rheumatoid arthritis, based on scientific trials that have shown that curcumin can decrease the production of these cytokines in the blood plasma [44]. Curcumin’s second method of averting cytokine formation involves changing the polarization of macrophages from M2 (anti‐inflammatory) to M1 (proinflammatory) [45].

#### 4.1.3. Role in COX‐2 and LOX Enzyme Activity

Proinflammatory mediators such as leukotrienes and prostaglandins are formed by the enzymes LOX and COX‐2 [46]. Prolonged inflammatory illnesses such as cardiovascular disease (CVD) and arthritis are linked to the overexpression of these enzymes. Curcumin directly inhibits LOX and COX‐2 activities. Curcumin overwhelms COX‐2 expression, which in turn decreases prostaglandin E2 (PGE2) levels, as per the investigations [47]. Furthermore, it also prevents leukotriene synthesis via the LOX pathway. Therefore, the dual inhibition of LOX and COX‐2 is helpful, especially when these mediators are present at higher levels in conditions such as asthma and inflammatory bowel disease [48].

#### 4.1.4. Supporting Evidence From Studies

In preclinical and clinical models, curcumin has been shown to decrease the levels of inflammatory markers. This is evident in the randomized controlled trial by Mohammadian et al. [49]; curcumin supplementation drastically decreased inflammation indicators, including the erythrocyte sedimentation rate (ESR) and C‐reactive protein (CRP), in patients with rheumatoid arthritis. It combines synergistic effects with natural medicines, such as ginger and other herbs, with similar suppression of inflammatory pathways, implying its potential use in combination therapy [50]. Mechanistic studies have indicated that the anti‐inflammatory activity of curcumin is augmented by its ability to chelate metal ions and scavenge free radicals, which further reduces inflammation caused by oxidative stress [51].

The interaction of curcumin with critical inflammatory signaling pathways makes it a potential therapeutic compound. Curcumin decreases the activity of COX‐2 and LOX enzymes, downregulates proinflammatory cytokines, and inhibits NF‐κB to target inflammation at different levels. These benefits are supported by preclinical and clinical data, making curcumin a potential therapeutic option for inflammatory diseases. More can be achieved regarding its bioavailability and therapeutic efficiency by researching improved formulations, such as nanoparticle distribution. Curcumin exerts profound and long‐lasting effects on inflammatory processes through the control of epigenetic mechanisms and its direct modification of inflammation through biochemical pathways. Epigenetic regulation refers to heritable variations in gene expression that do not entail changes in the DNA sequence. Therefore, curcumin exerts anti‐inflammatory effects by influencing critical epigenetic processes including DNA methylation, histone modification, and noncoding RNA regulation [47, 51].

#### 4.1.5. DNA Methylation

DNA methylation is a process by which the addition of methyl groups to cytosine residues in CpG islands often leads to silencing of gene expression. Such changes in DNA methylation deregulate inflammatory genes under numerous conditions such as cancer, diabetes, and rheumatoid arthritis, which cause chronic inflammation. It has been shown that curcumin interferes with DNA methylation by interfering with the activity of DNA methyltransferases (DNMTs). For instance, it can hypermethylate anti‐inflammatory genes and as a result, reverse their expression. Hussain et al. [48] reported that curcumin has the potential to reduce the production of proinflammatory genes such as TNF‐α and IL‐6 by triggering their methylation. These results indicate that curcumin can reduce inflammation by altering abnormal methylation.

#### 4.1.6. Histone Modification

Gene obtainability and chromatin structure are exaggerated by post‐translational histone modifications, including acetylation, methylation, and phosphorylation. Acetylation and dysregulation of heterochromatin methylation are related to the gene activation that occurs during long‐lasting inflammation. Curcumin eases the acetylation of anti‐inflammatory gene loci–specific histones by revealing inhibitory activity in contrast to histone deacetylase. Genes that are anti‐inflammatory, such as IL‐10, show increased expression. From the side‐to‐side hang‐up of histone acetylation at the supporters of proinflammatory genes, curcumin also overwhelms its expression too [43, 45].

Curcumin also affects histone methylation by regulating histone methyltransferases (HMTs). Edwards et al. [42] stated that curcumin overwhelms proinflammatory cytokine expression by decreasing H3K4 methylation, a marker of active transcription, at the gene loci that encode these cytokines.

### 4.2. Regulation of Noncoding RNAs

MicroRNAs (miRNAs) and long noncoding RNAs (lncRNAs) are important noncoding RNAs that regulate inflammatory reactions. This provides innovative opportunities for epigenetic regulation because curcumin controls these particles [45].

#### 4.2.1. miRNAs

Curcumin affects the regulation of miRNAs associated with inflammation. This was accomplished by inspiring the manifestation of miR‐146a, a miRNA recognized to overwhelm NF‐κB activation, which consequently results in the production of TNF‐α and IL‐6. Makuch et al. [45] reported that curcumin overwhelmed miR‐155, another activator of the proinflammatory pathway.

#### 4.2.2. Long Noncoding RNAs

Further investigation is needed to confirm the allegations that curcumin modifies lncRNAs that control inflammatory pathways. Curcumin also has the potential to regulate prolonged inflammation by directing the expression of long noncoding RNAs, which has an impact on the transcription of downstream inflammatory mediators [44].

#### 4.2.3. Inhibition of Inflammasome Activation

Inflammasomes, a group of proteins comprising IL‐1β and various proinflammatory cytokines, are also regulated by epigenetic regulators [52]. Curcumin overwhelms inflammasome initiation by altering chromatin accessibility of inflammasome component‐coding genes. Hasanzadeh et al. [53] reported that it overwhelms inflammation and NLRP3 inflammasome transcription by inhibiting the histone acetylation of the enzyme promoter. Through epigenetic intonation, curcumin can exert its anti‐inflammatory effects on several disorders: (1) Curcumin epigenetically alters epigenetic marks on joint inflammation–related genes, thus reducing the severity of the disease [49]. (2) Curcumin can modulate inflammation associated with insulin resistance by controlling histone modifications and miRNAs [48]. (3) Curcumin suppresses tumor‐promoting inflammation in cancer models by reducing proinflammatory mediators through DNA methylation and histone modifications [44].

Curcumin offers a definitive remedy for inflammatory disorders as it may modify the epigenetic pathways that control inflammation. Fasooto et al. [54] reported that curcumin can suppress proinflammatory gene expression through a multifaceted strategy that influences DNA methylation, histone modification, and noncoding RNAs. The therapeutic potential of this molecule may be further augmented by an ongoing investigation of its bioavailability and exact epigenetics. Owing to its ability to regulate cellular and systemic oxidative stress, curcumin has the potential to become the main drug for treating degenerative illnesses. Decreasing blood glucose levels relieves oxidative stress in diabetes and other illnesses, leading to the production of ROS [55]. Its antioxidant properties protect brain cells from oxidative damage and prevent neurodegenerative conditions, as reported by Ballester et al. [56]. Because curcumin persuades the Nrf2 and HO‐1 pathways, along with its direct capability to scavenge ROS, it serves as an operative therapeutic agent in contrast to oxidative stress. By supplementing endogenous antioxidant defenses and reducing ROS‐mediated impairment, curcumin appears to offer promising strategies for the anticipation and treatment of oxidative stress illnesses [33]. The therapeutic efficiency of curcumin may be augmented by further investigation of its absorption and the preparation of synergy‐based amalgamations.

### 4.3. Antioxidant Mechanisms and Oxidative Stress

Curcumin is a polyphenolic composite found in *C. longa* and is known to have strong antioxidant activity. One such method to bind oxidative stress is through the direct scavenging of ROS and by moderating antioxidant defense pathways, such as the Nrf2 pathway and overexpression of heme oxygenase‐1 (HO‐1). These processes are vital for diminishing cellular impairment and preventing oxidative stress‐related illnesses [57].

ROS include a range of naturally occurring products of cellular metabolism, including superoxide anions, hydroxyl radicals, and hydrogen peroxide. Overproduction of ROS results in oxidative stress, which is a major factor in the etiology of prolonged diseases such as diabetes, cancer, and cardiovascular illness [55]. According to Tuong et al. [58], free radicals are formed by curcumin through the neutralization of free radicals by scavenging ROS, whereby phenolic and β‐diketone clusters directly donate hydrogen atoms or electrons. As an extra step to steadying membranes and overwhelming lipid peroxidation, this procedure ceases the oxidation of proteins and DNA [59].

Furthermore, curcumin reduces the Fenton reaction, a technique that yields ROS by chelating metal ions such as copper and iron. Curcumin decreases the oxidative impairment in biological systems by inhibiting the generation of ROS formed by metals [60]. Curcumin has been experimentally proven to reduce ROS‐related toxicity in rats exposed to heavy metals such as cadmium [61]. Furthermore, curcumin combined with other bioactive substances increases its antioxidant potential. For instance, as per Silva et al. [62], combining *C. longa* with piperine improves ROS scavenging and further reduces oxidative stress in clinical situations, that is, in hemodialysis patients.

#### 4.3.1. Activation of Antioxidant Defense Systems

Through the modulation of key antioxidant pathways, curcumin stimulates cellular defense mechanisms to combat oxidative stress. A key pathway among these is the Nrf2 pathway. Nrf2 transcription factors control cellular redox homeostasis and oversee the expression of antioxidant enzymes [63].

#### 4.3.2. Nrf2 Pathway Activation

One inhibitor of Nrf2 is Keap1, an Nrf2 inhibitor that can be targeted by curcumin to alter its cysteine residues. Before it is produced, Nrf2 acquaintances with AREs on DNA initiate gene transcription to produce enzymes, such as catalase, glutathione peroxidase (GPx), and SOD [63]. By clearing ROS, such enzymes assist in regulating cellular redox balance [64]. Studies in sea bass and other aquatic species have shown that curcumin can effectively enhance antioxidant defenses under conditions of oxidative stress [65]. In a clinical trial of patients with Type 2 diabetes mellitus, oral curcumin supplementation (500 mg/day) upregulated Nrf2‐regulated antioxidant proteins, such as NQO1, and attenuated oxidative stress and inflammation markers, indicating activation of Nrf2‐mediated antioxidant defenses in human subjects [66].

#### 4.3.3. HO‐1 Expression

One of the cytoprotective enzymes induced by curcumin is HO‐1, which converts heme into carbon monoxide, biliverdin, and free iron. The antioxidant and anti‐inflammatory properties of these metabolites aid in decreasing oxidative damage and improving cellular repair [67]. Mitochondrial impairment or dysfunction is the main cause of oxidative stress–induced glitches. Studies in exhausting animal models have revealed that curcumin‐induced HO‐1 induction decreases this impairment [67].

#### 4.3.4. Synergistic Effects and Clinical Implications

In addition to scavenging ROS and inducing defense mechanisms, curcumin has antioxidant properties. It synergistically interacts with other natural antioxidants, such as vitamins C and E, to enhance overall redox stability [68]. In patients with chronic diseases such as kidney and cardiovascular disorders, curcumin treatment increases oxidative biomarkers and reduces inflammation based on clinical studies [69, 70].

Curcumin may control degenerative diseases by modulating oxidative stress at cellular and systemic levels. For instance, by regulating oxidative stress, curcumin reduces its levels in diabetes, wherein high blood sugar levels elevate ROS generation [55]. As it slows the onset of oxidative damage in brain cells, its antioxidant property helps prevent neurodegenerative diseases [56].

Curcumin has been described as a multifaceted therapeutic agent in the face of oxidative stress because of its various mechanisms of action as an antioxidant; these involve both its direct capabilities to scavenge ROS and stimulate the Nrf2 and HO‐1 pathways [71]. Curcumin is a possible approach for preventing and managing disorders involving oxidative stress through the potentiation of endogenous antioxidant defenses while lowering ROS‐related damage. An even greater therapeutic potential of curcumin could be developed from future absorption studies of curcumin and synergistic studies.

#### 4.3.5. Prevention of Lipid Peroxidation and DNA Damage

*C*. *longa*, particularly its active component curcumin, has been extensively studied for its potent antioxidant properties, particularly its ability to inhibit lipid peroxidation and protect against DNA damage. Most of the diseases associated with degenerative conditions occur during these processes. Curcumin provides a wide range of ways to protect cellular lipids and DNA, with subsequent risks of diseases being reduced through lowered oxidative stress.

#### 4.3.6. Prevention of Lipid Peroxidation

Lipid peroxidation is a process by which lipids, especially polyunsaturated fatty acids, are attacked by ROS, leading to the production of lipid peroxides. This can hinder cellular activity, seriously harm cellular membranes, and accelerate the development of many diseases [67]. Curcumin is a strong antioxidant that prevents lipid peroxidation in several ways.

#### 4.3.7. Direct Scavenging of ROS

Curcumin directly scavenges ROS, which are the primary cause of lipid peroxidation. Curcumin prevents free radicals, such as superoxide and hydroxyl radicals, from damaging lipids on the cell membrane by neutralizing them [59]; Therefore, it conserves the integrity of cellular membranes by reducing oxidative damage to the lipid bilayer.

#### 4.3.8. Inhibition of Lipid Peroxidation Pathways

Curcumin has been shown to inhibit the formation of key lipid peroxidation enzymes such as COXs and lipoxygenases. Curcumin prevents the overproduction of lipid peroxides and other dangerous oxidation products by downregulating these enzymes [64].

#### 4.3.9. Modulation of Antioxidant Enzymes

Curcumin also increases the activity of natural antioxidant enzymes such as GPx, catalase, and SOD, which scavenge ROS and suppress lipid peroxidation. For example, Tuong et al. [58] reported that curcumin regulates the generation of these enzymes through the activation of the Nrf2 pathway. This antioxidant resistance network is essential for avoiding lipid impairment in cells.

#### 4.3.10. Inhibition of Fenton Reaction

The Fenton reaction produces hydroxyl radicals from hydrogen peroxide in the presence of Fe^2+^, thereby initiating lipid peroxidation. According to a previous study [59], curcumin can inhibit lipid peroxidation and damage inflicted on cells through the inhibition of the process. The antioxidant action of curcumin against lipid peroxidation has been recognized in numerous circumstances, ranging from cell culture in vitro experiments to prototypes in vivo in animals. For instance, as per Ballester et al. [56], curcumin treatment significantly reduced the levels of malondialdehyde (MDA), a well‐known marker of lipid peroxidation, in rat models of oxidative stress.

#### 4.3.11. Prevention of DNA Damage by Curcumin

DNA damage, particularly oxidative damage, is involved in the etiology of most diseases. The development of DNA adducts, base modifications, and strand breaks are DNA scratches that can cause ROS. Mutations, genomic uncertainty, and cell death can occur due to these lesions if they are not cured. Curcumin protects DNA from oxidative damage using various methods.

#### 4.3.12. Scavenging of ROS and RNS

A common DNA defensive mechanism of curcumin is its ability to scavenge ROS and RNS. Curcumin decreases the risk of oxidative DNA damage by preventing ROS and RNS from interacting with the DNA. An oft‐measured indicator of oxidative DNA damage and DNA strand smashing, 8‐oxo‐2′‐deoxyguanosine (8‐oxodG), was significantly repressed by curcumin treatment [72].

#### 4.3.13. Enhancing DNA Repair Mechanisms

Experts have reported that curcumin improves the production of DNA repair proteins, particularly those that contribute to the NER and BER pathways. Sathyabhama et al. [67] stated that curcumin improves the DNA repair capability, indicating that it certifies genomic integrity and constrains the accretion of mutations.

#### 4.3.14. Induction of Antioxidant Enzymes

In addition to hindering DNA impairment, curcumin excites antioxidant enzymes such as catalase and glutathione S‐transferase (GST). In addition to detoxifying harmful constituents, enzymes protect DNA from oxidative stress. By endorsing these enzymatic systems, curcumin indirectly decreased DNA impairment indirectly [65].

#### 4.3.15. Anti‐Inflammatory Effects

Amplification of DNA impairment by inflammatory action is recurrent, and inflammatory cytokines are responsible for the generation of ROS. Curcumin reduces inflammation and prevents ROS‐induced DNA damage induced by chronic inflammation [55]. According to Asanga et al. [73], curcumin can protect DNA and downregulate mediators of inflammation, such as TNF‐α and IL‐6, while reducing inflammation.

#### 4.3.16. Reduction of Mutagenic Effects

Curcumin has also been shown to reduce the harmful effects of various chemical and environmental insults, including carcinogens, by simply altering cellular reactions to these substances [74]. This involves reducing the levels of DNA adducts and mutations caused by chemicals including tobacco smoke and aflatoxin [67].

#### 4.3.17. Prevention of Cancer Initiation

Curcumin protects against oxidative DNA damage and promotes cancer onset. It inhibits the expression of genes that code for the DNA response to damage, thereby suppressing oncogene activation, which is crucial for the anticancer activity of curcumin, as deduced from several studies [55].

**FIGURE 2:**
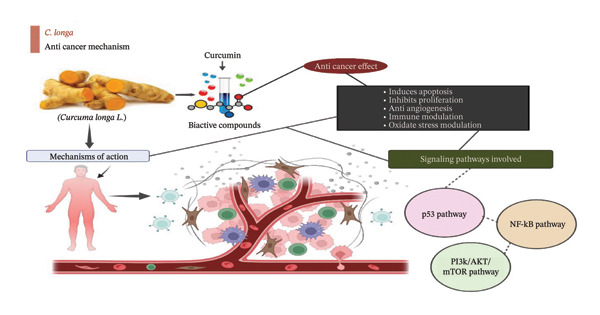
Anticancer mechanism of action of turmeric. The major anticancer effects, including the induction of apoptosis and inhibition of cell proliferation, mediated through the regulation of key molecular pathways such as p53, NF‐κB, PI3K/Akt, and mTOR.

Curcumin is an active natural agent that exhibits preventive effects against oxidative stress–related disorders, including cancer, neurological diseases, and CVDs and may inhibit lipid peroxidation and DNA damage. The antioxidant properties of curcumin are due to the scavenging of ROS, enhancement of endogenous antioxidant defenses, and modification of DNA repair pathways to protect cellular lipids and DNA. The protective properties of curcumin in lowering DNA damage and lipid peroxidation make a strong case for its potential as a treatment to fight oxidative stress and delay the onset of chronic illnesses [73]. Figure 2 summarizes the anticancer mechanisms of turmeric, involving apoptosis induction, proliferation inhibition, and modulation of the p53, NF‐κB, PI3K/Akt, and mTOR pathways.

## 5. Molecular Crosstalk With Gut Microbiota

Curcumin, a bioactive phytochemical present in *C. longa*, has been studied to gain insights into its action on microbiota composition and therapeutic potential in the gut [75]. This is because curcumin’s interaction with gut microbiota controls its bioactivity and how strongly it impacts human health; curcumin also possesses antioxidant, anti‐inflammatory, and immunomodulatory properties. Experiments have shown that curcumin significantly alters the gut flora. Human digestion, the immune system, and metabolism are facilitated by various bacterial communities in the gut. Through the modification of the gut microbiota, curcumin may facilitate the growth of beneficial bacteria or inhibit the proliferation of harmful bacteria, thereby safeguarding gut health and inflammation. Curcumin has been found to modify the gut microbiota. This is achieved by decreasing the number of pathogenic bacteria, such as *Proteobacteria* and *Firmicutes* and increasing the number of beneficial bacteria, such as *Bifidobacterium* and *Lactobacillus* [31, 75].

These modifications are important because cancer, metabolic diseases, inflammatory diseases, and other health conditions are associated with the composition of the microbial communities [76]. More importantly, curcumin supports the hypothesis that the gut microbiota is involved in mediating the therapeutic activity of curcumin, since it is known to control microbiota composition. It controls microbial metabolism and affects microbiota composition. Gut microbes facilitate the biotransformation of curcumin into active compounds that exert additional effects on the host health. The bioavailability of curcumin metabolites is greater than that of curcumin, and curcumin can exert potent antioxidant and anti‐inflammatory effects [77].

This process reflects the biphasic interaction between curcumin and the gut microbes, demonstrating its extensive medicinal potential. Curcumin has a few conventional biomedical applications because of its low bioavailability, even though it is bioactive. Curcumin is acted upon by gut bacteria to metabolize it further into more effective metabolites that increase its bioactivity, a recent study affirms. Memarzia et al. [43] discovered that curcumin is absorbed, metabolized, and distributed by gut microbiota to increase its potential health benefits. The generation of bioactive metabolites and curcumin breakdown by microbes are two primary pathways through which their bioactivity is increased. Intestinal microbial enzymes drive the uptake of THC metabolites into the bloodstream. There have been propositions that the antioxidant and anti‐inflammatory capacities of these metabolites are even greater than those of their parent compounds [30].

By facilitating the bioconversion of curcumin to its active form, the microbiota enhances drug efficacy and system bioavailability. It is likely that the pharmacokinetics of curcumin are influenced by gut microbiota. Curcumin can enhance enteric absorption and retard breakdown by interacting with the enteric bacteria. Therefore, the health status of the microbiota plays an important role in determining the efficacy of the curative actions of curcumin [31]. Table 3 summarizes the molecular crosstalk between curcumin and gut microbiota and its influence on microbial composition and host physiological functions.

**TABLE 3:** Molecular crosstalk with gut microbiota and curcumin’s influence on gut microbiota composition.

Topic	Details	References
Curcumin’s influence on gut microbiota composition	Curcumin has been found to alter the gut microbiota in various ways. It can enhance the abundance of beneficial microbiota species while decreasing harmful ones.	[43]
	Curcumin modulates microbiota through its anti‐inflammatory and antioxidant properties, leading to improved gut health.	[47]
	Studies indicate that curcumin can reduce gut permeability, promote a healthy microbiome balance, and protect against gut‐related diseases.	[76]
	Curcumin’s ability to influence the microbiota composition has implications for inflammatory bowel diseases, as it reduces pathogenic bacteria and supports gut homeostasis.	[44]

Role of microbiota in enhancing curcumin bioactivity	The gut microbiota can impact the bioavailability and effectiveness of curcumin by facilitating its metabolism into bioactive metabolites, thus enhancing its therapeutic effects.	[43]
	Gut microbiota can convert curcumin into metabolites such as tetrahydro curcumin, which may be more bioactive than curcumin.	[77]
	Microbial enzymes such as β‐glucuronidases play a significant role in increasing curcumin’s bioactivity through deglucuronidation.	[47]
	The interaction between curcumin and gut microbiota enhances the anti‐inflammatory and antioxidant effects, contributing to its therapeutic potential.	[48]
	Microbiota‐driven modification of curcumin boosts its systemic availability and potency, which could be key to therapeutic applications in diseases like cancer and arthritis.	[51]

### 5.1. Curcumin and Gut‐Derived Metabolites

The anti‐inflammatory properties of curcumin could be greatly increased by its interaction with the gut flora, as shown in a recent study [78]. The ability of the gut microbiota to metabolize and produce compounds that could influence inflammatory pathways is where curcumin exerts its anti‐inflammatory effects. For example, Xu et al. [29] found that curcumin metabolites, particularly those converted by gut bacteria, restrict both the production of proinflammatory cytokines and control of the NF‐κB, a primary element of inflammation. Furthermore, research has indicated that curcumin modulates systemic inflammation by altering the gut–liver axis through microbiota‐mediated metabolism. The direction of the inflammatory processes and immune responses is crucial for prolonged conditions, metabolic syndrome, cardiovascular illness, and autoimmune conditions. This axis is vital to the procedure. Hassan et al. [79] reported that curcumin can cause the body to react more efficiently to inflammation by altering the activity of microbes and forming composites that reduce inflammation. Curcumin constrains the microbiota from manufacturing SCFAs such as butyrate, which is a noteworthy interface. The anti‐inflammatory activity of SCFAs is a consequence of the fermentation of dietary fiber by the gut microbiota. Meanwhile, curcumin improves the production of SCFAs, conserves the reliability of the gut barrier, and decreases systemic inflammation.

Qin et al. [80] highlighted the complicated interface between diet, gut microbiota, and the regulation of inflammation through the collaboration between curcumin and metabolites formed from the gut. A significant factor in improving curcumin bioactivity, predominantly in the management of inflammation, is the molecular link between curcumin and the intestinal flora. Curcumin expands the development of beneficial microbes in the gut, overwhelms the growth of damaging microbes, and rouses the synthesis of its bioactive metabolites, with a considerable effect on its therapeutic activity [81]. In addition to cumulative curcumin bioavailability and effectiveness, this mutually advantageous relationship draws consideration to the critical function the gut microbiota plays in adaptable inflammatory illnesses and overall health. The anti‐inflammatory and antioxidant properties of curcumin and its ability to influence gut flora provide a holistic method to treat prolonged diseases. More curcumin‐based treatment methods will likely emerge, as further investigation leads to the relationship between this bioactive composite and gut microbiota.

## 6. Applications of Curcumin in Chronic Diseases

A large portion of the scientific attention on curcumin, usually recognized as *C. longa*, comes from its supposed therapeutic usefulness for a myriad of prolonged illnesses, particularly those concerning inflammatory etiology. Prolonged inflammation is an amalgamating element in most illnesses, with prolonged diversity, and is a prerequisite for pathogenesis [82]. The anti‐inflammatory, antioxidant, and immunomodulatory properties of curcumin make it a promising drug for the treatment of several diseases.

### 6.1. CVDs

Atherosclerosis, endothelial dysfunction, and the resultant cardiovascular trials have all been linked to inflammation. CVDs account for a disproportionate percentage of mortality and morbidity worldwide. As it improves oxidative stress, arouses vascular injury, and eases arterial plaque formation, prolonged inflammation is the main component of CVD pathogenesis. The consequences of curcumin in the cure and prevention of cardiovascular conditions have been encouraging because of its anti‐inflammatory properties. One of the main players in the development of atherosclerosis, curcumin, was reported by Hu et al. [63] to moderate oxidative stress. Endothelial dysfunction is one of the main risk factors for cardiovascular illnesses and can relieve this disorder by decreasing ROS and augmenting the activity of antioxidant enzymes. The ability of curcumin to overwhelm proinflammatory cytokines such as TNF‐α and IL‐6 maximizes its cardiovascular defensive effects [67]. In a trial involving 59 healthy adults, 200 mg of curcumin administered for 8 weeks resulted in an approximately 3.0% increase in flow‐mediated dilation (FMD) compared to placebo (this represents an important improvement in the function of the endothelium) [83]. In 39 healthy middle‐aged and older adults, 12 weeks of curcumin (2000 mg/day Longvida®) resulted in resistance artery flow responses increasing by 37% and brachial artery FMD increasing by 36% in comparison with placebo and baseline. This shows an improvement in vascular dilation mediated by NO [84].

The antioxidant and anti‐inflammatory properties of curcumin have been investigated in the context of cardiovascular risk factors in experimental settings. For instance, supplementation with *C. longa* extract enhances endothelial function and prevents markers of oxidative stress in healthy subjects, as described by Hajleh and Al‐Dujaili [70]. Based on these results, curcumin could be useful in anticipating cardiovascular illnesses in both deceptively healthy subjects and those previously diagnosed with the disorder.

### 6.2. Neurodegenerative Diseases

Neurodegenerative illnesses such as AD, PD, and multiple sclerosis (MS) have been associated with prolonged neuroinflammation. For example, inflammation contributes to the formation of amyloid‐beta plaques and tau tangles, the pathophysiology of which causes neuronal damage and cognitive decline in Alzheimer’s disease. Several studies have examined the effect of curcumin on neuroinflammation. Curcumin has been shown to offer neuroprotection by blocking the activation of microglial cells, which play an essential role in neuro‐inflammation. According to a study published by Park et al. in 2021, curcumin decreased oxidative stress and inflammatory responses in cadmium‐poisoned animal models associated with neurodegenerative processes. It can also be used to treat AD owing to its permeability across the blood–brain barrier and its modulatory effect on the CNS. Through interference with important signaling pathways such as NF‐κB, curcumin can inhibit neuroinflammation owing to the reduced production of proinflammatory cytokines in the brain [29].

Clinical trials have shown that curcumin supplementation has promising benefits for patients through clinical trials. A meta‐analysis involving nine randomized controlled trials (with 501 participants) demonstrated that curcumin supplementation had a favorable impact on global cognition (SMD ≈ 0.82) compared to placebo. This impact was more pronounced in older adults (≥ 60 years) and with an intervention duration of 24 weeks or more [85].

By lowering oxidative stress and biomarkers of inflammation, curcumin improves cognitive functioning in patients with mild to severe disease, as reported in a study by Memarzia et al. [43]. Such outcomes establish the validity of curcumin as a potential adjuvant therapy for neurodegenerative diseases, primarily for those with inflammatory components [43].

### 6.3. Autoimmune Disorders

The hallmark of autoimmune diseases is the dysregulated activation of the immune system, which is responsible for tissue damage and persistent inflammation [86]. For example, the immune system mistakenly attacks synovial joints in RA, leading to chronic pain, inflammation, and joint deformities [87].

Several studies have demonstrated the immunomodulatory potential of curcumin for the treatment of autoimmune disorders. Curcumin has been shown to moderate the immune system by decreasing the production of proinflammatory cytokines and suppressing the initiation of immune cells such as macrophages, B‐cells, and T‐cells. Aggarwal et al. [31] reported that the synthesis of NF‐κB was repressed by curcumin. A critical feature in the pathogenesis of autoimmune diseases such as RA is that this transcription feature contributes to the body’s inflammatory response. It is possible that curcumin hinders NF‐κB initiation, which in turn reduces inflammation and hinders the progression of joint impairment in patients with RA. Frequent scientific investigations have focused on the effectiveness of curcumin in the treatment of autoimmune diseases.

An investigation by Joshi et al. [88] showed that RA patients treated with curcumin showed a noteworthy decrease in systemic inflammatory parameters, such as ESR and CRP. As a supernumerary of conformist disease‐modifying antirheumatic drugs (DMARDs), curcumin has also been shown to decrease pain and improve the quality of these patients’ joints. In a meta‐analysis on rheumatoid arthritis, curcumin supplementation showed a greater reduction in the ESR (SMD ≈ −3.72) and CRP (SMD ≈ −2.91) than placebo, suggesting that there is a considerable anti‐inflammatory effect on systemic inflammatory markers in rheumatoid arthritis patients [89].

The antioxidant effect of curcumin in reducing oxidative stress has also been reported in autoimmune diseases. For example, curcumin can diminish oxidative stress and cognitive dysfunction induced by Cd exposure [68]. In autoimmune illnesses of the brain such as MS, this is vigorous.

### 6.4. Mechanistic Insights and Case Studies

The therapeutic effects of curcumin in prolonged inflammatory illnesses can be mechanistically attributed to its multifaceted mode of action on significant signaling pathways [90]. Curcumin is thought to decrease the worldwide inflammatory load in prolonged diseases by constraining the initiation of inflammatory mediators such as COX‐2, LOX, and other cytokines [91].

The antioxidant action of curcumin is vital to its beneficial actions in frequent illnesses and situations such as CVD, neurological disease, and autoimmune illness. Cases reported on the use of curcumin in treating prolonged disorders are additional indications of its therapeutic effectiveness. With a medical trial populace of patients predisposed to cardiovascular illness, Hajleh and Al‐Dujaili [70] exposed that curcumin supplementation importantly enhanced endothelial function while dropping oxidative stress. A population of patients with RA has also experienced developments in joint movement, pain, and inflammation following the ingestion of curcumin [43].

These case reports support the use of curcumin for the treatment of illnesses with prolonged inflammation. Curcumin is a prospective treatment for prolonged inflammatory illnesses, with antioxidant, immunomodulatory, and anti‐inflammatory effects. Numerous clinical and experimental studies have established methods to efficiently decrease oxidative stress and inflammation. Several studies have focused on its potential application in the treatment of autoimmune, cardiovascular, and neurological diseases. Curcumin has outstanding promise, but additional investigation is required to discover its precise mechanisms of action and the optimal dosing and treatment of diverse patient assemblies. Curcumin can be a harmless natural substitute for modern medications in numerous patient populations, and additional investigations will make it an appreciated tool for prolonged disease administration.

## 7. Therapeutic Potential of Curcumin: Advancements in Future

Impending curcumin derivative nutraceuticals, current progress in curcumin delivery, and knowledge breaches are deliberated in this section of the conversation of future directions for curcumin investigation. The squat bioavailability of curcumin is an important barrier for its therapeutic use. Owing to its rapid metabolism, lipophilicity, and poor water solubility, curcumin is rapidly released from the body as it arrives [29]. Experts have been engaged in the development of novel drug delivery systems to improve the bioavailability and effectiveness of curcumin. New developments in delivery preparations include nanoparticles, micelles, and liposomes. These mechanisms not only improve curcumin solubility and consistency but also improve its delivery to mark tissues [92]. For example, Fu et al. [22] reported that curcumin‐encapsulated nanoparticles advance the pharmacokinetic profile of the composite, resulting in increased levels of curcumin in circulation and enhanced anti‐inflammatory and antioxidant activities. One of the promises of neurodegenerative illness, including Alzheimer’s, is the contest for bringing drugs to precise organs; curcumin in a micelle arrangement could be supportive of this [93]. Furthermore, investigations on the interface of curcumin with other materials, including piperine (a black pepper constituent), have improved its bioavailability. Hussain et al. [48] stated that this method seeks to resolve the problem of curcumin bioavailability in a multifaceted manner.

### 7.1. Curcumin‐Based Nutraceuticals

Curcumin derivative nutraceuticals have immense potential as natural, low‐cost supplements for the treatment or prevention of prolonged inflammatory diseases. Owing to its strong anti‐inflammatory, antioxidant, and anticancer activities, curcumin is a feasible dietary supplement. This marks all the cellular, enzymatic, and molecular levels. Curcumin, as an adjuvant agent for illnesses, such as cancer, cardiovascular illnesses, and rheumatoid arthritis, has been investigated in numerous experimental trials [94].

**FIGURE 3:**
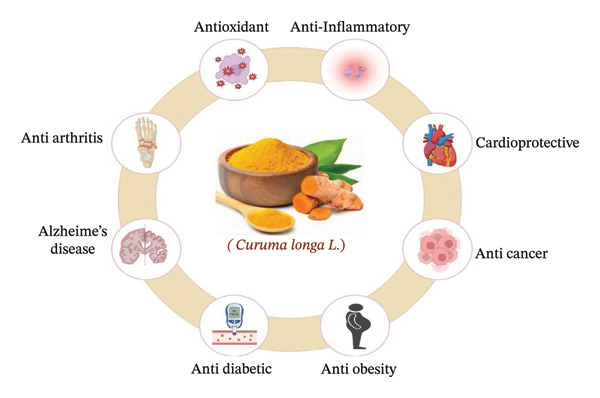
Therapeutic potential of turmeric in arthritis, autoimmune diseases, cardiovascular disorders, and antioxidant‐related conditions.

Curcumin‐based nutraceuticals have been shown to reduce inflammatory markers, including interleukins and CRP, making them a useful supplement to conventional treatments [88]. However, improvements in curcumin formulations are needed to reach full therapeutic potential in clinical settings. Studies have shown that the clinical utility of curcumin can be significantly increased by using improved delivery mechanisms, even if curcumin in its natural form is not always associated with the desired clinical results. Moreover, future directions for curcumin‐based nutraceuticals that formulate an individual’s specific genetic profile and pathophysiology of their disease could emerge from the rapidly expanding personal medicine movement (Figure 3) [92]. Figure 3 summarizes the therapeutic potential of turmeric in arthritis, autoimmune diseases, cardiovascular disorders, and antioxidant‐related conditions.

### 7.2. Synergistic Effects of *C. longa*

It is widely known that the primary bioactive compound curcumin, which is present in *C. longa*, possesses potent anti‐inflammatory properties. Due to the novel molecular mechanisms of curcumin and the complex interaction between the whole‐plant extract and *C. longa*, these two molecules have synergistic activities. In addition to its ability to control significant inflammatory pathways, such as NF‐κB, COX‐2, and proinflammatory cytokines, *C. longa* contains other curcuminoids and volatile oils that enhance the stability and bioactivity of curcumin [4].

The complete *C. longa* matrix enhanced the pharmacokinetic profile of curcumin, as reported by Abd El‐Hack et al. [8], providing a broader set of biological effects than curcumin itself. The anti‐inflammatory activity, systemic absorption, and bioavailability are increased by such synergy, which does not occur when curcumin is taken up by itself [8]. In addition, various formulations involving the blending of *C. longa* and curcumin have exhibited improved effectiveness in mitigating oxidative stress and chronic inflammation [94].

According to Aggarwal et al. [94], nanoformulations of curcumin have a more intensive binding capacity in inflammatory areas and inhibit inflammatory mediators such as TNF‐α, IL‐6, and iNOS for a longer duration. This is supported by research conducted by Akaberi, Sahebkar, and Emami [4], which revealed that *C. longa*‐derived chemicals have a dual anti‐inflammatory mechanism through the concurrent suppression of both the lipoxygenase and COXs pathways. *C. longa* and its bioactive constituent, curcumin, act synergistically as promising adjunct agents for inflammatory disorders, especially when formulated to enhance bioavailability and therapeutic efficacy.

### 7.3. Research Gap

However, many unanswered questions remain regarding curcumin. Although the results from preclinical and clinical studies are encouraging, much is known, especially regarding the long‐term safety and efficacy of curcumin in chronic disease populations. Curcumin is considered relatively safe; however, its long‐term use in humans is rarely known, and most studies are short‐term or use animal models [31].

Long‐term clinical trials are required to identify the best dosage plan, possible adverse effects, and combinations with other drugs or supplements. Another crucial area that requires further research is the identification of molecular targets and mechanisms of action of curcumin in many illness models. Although curcumin has been shown to modulate several significant inflammatory pathways, including MAPKs and NF‐κB, how curcumin works at the cellular and molecular levels [93]. Further research is needed to determine whether curcumin plays a role in regulating gut microbiota, which has been associated with various chronic diseases, and its impact on intestinal health and systemic inflammation [77].

Curcumin may be interrelated with other bioactive particles, dietary supplements, or adjuvant rehabilitation; this possibility should be examined in future studies. This information can provide insights into the use of curcumin as a part of amalgamation treatments for polydiseases by emphasizing how curcumin interacts with other medications, mainly traditional pharmacological drugs [79]. Neurodegenerative and autoimmune conditions may result in new curcumin treatments if the action of curcumin on microbiota and immune cells is discovered [94].

Curcumin has enormous therapeutic value in prolonged inflammatory diseases. However, various clinical applications and delivery optimization bottlenecks must be overcome. New advancements in curcumin delivery include micelles and nanoparticles, which can target the tissues of interest and enhance their bioavailability. The vast field of curcumin‐based nutraceuticals hold tremendous potential for improving health outcomes and should be combined with conventional treatments. There is still a considerable need for studies to fill knowledge gaps regarding the long‐term safety of curcumin, its molecular mechanisms, and its therapeutic applications. Therefore, in the future, a detailed multidisciplinary approach is necessary to fully understand the medicinal potential of curcumin.

## 8. Conclusion and Future Trends

Curcumin is the main bioactive compound found in *C. longa*. It has significant anti‐inflammatory and antioxidant properties; hence, it may be considered a treatment for various chronic conditions. Curcumin reduces systemic inflammation and oxidative stress by scavenging ROS and regulating key inflammatory pathways including NF‐κB, COX‐2, and LOX. However, new opportunities for curcumin, namely, its impact on gut microbiota composition, have emerged regarding its potential cooperation with the same metabolites.

Encouraging the clinical use of curcumin, issues such as low bioavailability and the need for more comprehensive clinical trials have yet to be addressed. As research progresses, curcumin‐based treatments are likely to play a significant role in chronic inflammatory disorder management, offering a natural alternative to traditional treatment approaches. In the near future, further research on curcumin will achieve its true medicinal value by improving its drug delivery systems. Liposomal encapsulation, nanoparticle‐based formulations, and the addition of adjuvants, such as piperine, are novel approaches expected to alleviate curcumin bioavailability due to poor solubility and fast metabolism. These developments are expected to significantly improve the distribution and absorption of curcumin, thereby enhancing its efficacy in the treatment of inflammatory illnesses. Moreover, the investigation of the synergistic effects of curcumin with other bioactive substances, prescription drugs, or lifestyle changes may lead to new therapeutic approaches, particularly, for chronic illnesses with complex etiologies. Despite encouraging preclinical and short‐term clinical findings, several critical research gaps remain in the literature. Foremost among these is the lack of long‐term clinical trials evaluating safety, sustained efficacy, optimal dosing strategies, and population‐specific responses. In addition, although curcumin is known to influence multiple signaling pathways, its precise molecular targets and cell‐type–specific mechanisms of action remain incompletely understood and warrant further investigation.

Future research should also focus on how curcumin affects the functioning of immune cells and their interaction with the gut microbiota, as this may lead to new applications for treating inflammatory bowel disease and autoimmune illnesses. Long‐term clinical trials focusing on the safety, ideal dosage, and customized treatment plans are required to prove the effectiveness of curcumin in various populations. With advances in research, nutraceuticals based on curcumin are likely to become the new norm in clinical settings, offering an alternative or supplement to traditional medicines, primarily for illnesses for which there is no effective treatment. The fact that curcumin can be tailored to each patient’s unique genetic profile may enhance its therapeutic effect, in light of the trend in precision medicine. Hence, establishing curcumin as an essential factor in the management of chronic inflammatory disorders is feasible only through constant innovation and serious scientific studies.

## Author Contributions

Sammra Maqsood: writing–original draft preparation; writing–review and editing; methodology; conceptualization; Farhang Hameed Awlqadr: writing–review and editing; data curation; Iffat Ullah: writing–review and editing; formal analysis; Muhammad Tayyab Arshad: writing–original draft preparation; writing–review and editing; project administration; Humaira Parveen: writing–review and editing; conceptualization; Sayeed Mukhtar: writing–review and editing; visualization; Emmanuel Laryea: writing–original draft preparation; writing–review and editing; supervision.

## Funding

No funding was received for this manuscript.

## Disclosure

All authors have read and approved the final manuscript.

## Conflicts of Interest

The authors declare no conflicts of interest.

## Data Availability

The data that support the findings of this study are available from the corresponding author upon reasonable request.
